# Integrating levels of explanation: a Grand Challenge for the next decade of cognitive science

**DOI:** 10.3389/fpsyg.2026.1860962

**Published:** 2026-07-01

**Authors:** Eddy J. Davelaar

**Affiliations:** School of Psychological Sciences, Birkbeck, University of London, London, United Kingdom

**Keywords:** algorithmic level, cognitive science, computational level, Grand Challenge, implementational level, levels of analysis

## Introduction

Cognitive science has long been defined by its ambition to explain the mind across multiple levels of analysis. This multilevel perspective is widely recognized as a defining strength of the field and is reflected in a wealth of innovative and influential research spanning perception, memory, language, decision-making, learning, reasoning, philosophy, sociology, and artificial intelligence. However, despite this progress, genuine integration across these levels remains limited. As empirical methods diversify and theoretical approaches proliferate, there is an increasing risk of fragmentation. At the same time, these developments have renewed attention to fundamental questions about how cognitive science should conceptualize, study, and explain cognition. In this Grand Challenge article, I propose four interrelated challenges, grounded in Marr's (1982) levels of analysis, that will shape the future trajectory of cognitive science. Together, these challenges address a central problem: how to build cumulative, generalizable, replicable, and societally relevant theories of cognition.

## Challenge 1: integrating levels of explanation

A long-standing challenge in cognitive science concerns the integration of explanations across multiple levels, ranging from neural mechanisms to computational principles, cognitive processes, behavior, and social context. Classic frameworks, such as Marr's (1982) distinction between computational, algorithmic, and implementational levels, have clarified the importance of multiple explanatory perspectives; however, practical integration across these levels remains elusive. Advances in neuroimaging and large-scale neural data have not always translated into corresponding advances in cognitive theory, while computational models often succeed in terms of performance without offering psychologically interpretable explanations.

What does it mean to integrate across multiple levels? The answer lies in using a methodological approach that goes beyond simply accumulating data at different levels. In the context of formal modeling, cross-level integration would mean developing models that are constrained by behavioral data, informed by neural mechanisms (not just principles), and capable of explaining cognitive function across tasks and contexts. Thus, it requires developing theories that meaningfully link the different levels.

Progress is essential for avoiding both reductionism and fragmentation within cognitive science. Programmes such as cognitive architectures (Langley et al., 2009) provide strong foundations for pursuing cross-level integration (Petersen and Sporns, 2015). Recent work in this area pursues integration with large language models (e.g., Wu et al., 2025) or other hybrid systems (González-Santamarta et al., 2025). Given the range of architectures, one might wonder whether a unified model of cognition is possible in principle. Under the assumption that there is a model that everyone can agree on, can this model explain the computational level of our mind? What is the human mind designed to compute or optimize? Candidate computational goals include utility maximization, predictive accuracy, bounded optimization, and even evolutionary fitness. The first Grand Challenge looks at the cross-level integration to answer the computational-level question of what human cognition is for.

## Challenge 2: bridging laboratory models and real-world cognition

Much of cognitive science relies on highly controlled experimental paradigms that trade ecological validity for internal validity. While this approach has yielded foundational insights, concerns have grown regarding the extent to which laboratory findings generalize to cognition as it occurs in naturalistic settings. Real-world cognition is dynamic, embodied, socially situated, and embedded in rich cultural and technological environments (Barsalou, 2008; Clark, 1997).

This challenge is compounded by the heavy reliance on WEIRD (Western, Educated, Industrialized, Rich, and Democratic) populations, which limits the universality of many cognitive theories (Henrich et al., 2010). A central challenge for the field is therefore to develop methods and theoretical frameworks that retain experimental rigor while capturing the complexity of real-world cognition. This addresses the various constraints placed on the cognitive system while it fulfills its computational-level goal. Advances in using virtual environments, naturalistic stimuli, and cross-cultural research provide promising avenues, but their integration into mainstream cognitive theory remains incomplete.

A recommendation for this challenge is to include a section on Ecology in manuscripts that introduce new theories or models. In this section, authors could discuss the sensitivity of their theory/model to background context, such as the language used, the semantic network of words and concepts, the statistical distribution of visual features in the environment (e.g., rural vs. urban), and so on. This would help to situate the work within the correct ecology and identify implicit assumptions that could lead to the misinterpretation of instructions or showing poor task performance due to a lack of prior exposure to certain visual statistics (e.g., vertical vs. horizontal). This addition may spur readers to replicate and conduct further research, thereby increasing our understanding of how different types of ecology influence cognitive performance and constrain cognitive architecture.

## Challenge 3: theoretical fragmentation and the need for integration

Cognitive science is characterized by a diversity of theoretical frameworks, including symbolic, connectionist, Bayesian, predictive processing, embodied, and enactive approaches. This pluralism has been intellectually productive, but it has also led to theoretical fragmentation, with competing frameworks often addressing similar phenomena using incompatible assumptions and vocabularies.

At an algorithmic level, editorial requirements to present new models in order to get published may have nudged us in this direction. To counter the further fragmentation of the theoretical progress, we should reward computational meta-analyses, where the mechanisms used in computational models are tested to establish whether they are necessary to account for cognitive performance. This can be done at a theoretical level (e.g., Estes, 1999) or through formal modeling (e.g., switching competing algorithms on or off within a general model framework). Such efforts are likely to result in composite models with only the necessary algorithms.

This third challenge encourages us to move beyond parallel theoretical silos and toward integrative or meta-theoretical perspectives that clarify how different approaches relate to one another. This does not eliminate theoretical diversity but rather develops principled ways to compare, combine, or delimit theories. Through such integration, empirical progress becomes cumulative, rather than risks remaining local.

## Challenge 4: expanding the boundaries of cognition

Finally, cognitive science faces a conceptual challenge concerning the boundaries of cognition itself. Traditional approaches have focused primarily on individual adult humans; however, growing evidence suggests that cognition is distributed across brains, bodies, artifacts, social groups, and cultures (e.g., Hutchins, 1995; Theiner et al., 2010). At the same time, advances in artificial intelligence and human-machine interaction raise new questions about how cognitive processes are shared, augmented, or transformed by technology.

Implementation-level analysis may not seem pertinent to cognitive scientists. However, the discussion on whether cognition can be implemented in a system that is not based on neurones is becoming increasingly relevant, as it is linked to the question of whether consciousness emerges from a particular physical substrate or from a particular type of information processing. This level of analysis invites scholars from biology, sociology, philosophy, mathematics, and physics to contribute to a fundamental question about cognition and intelligence. Needless to say, this discussion was initiated during the Macy conferences of the 1940s and 1950s and continues to this day. The fourth challenge is to navigate the implementation-level analysis in a world where some commentators have suggested that large language models exhibit properties resembling aspects of self-representation or metacognition (this remains a contested assertion). Cognitive science is the right interdisciplinary field to discuss whether human cognition, with its computational-level goals, can have different physical realizations. If not, then the “cognition” seen in other physical substrates (e.g., silicon) is not the same as human cognition. Its non-human cognitive architecture may very well solve a different computational-level problem (using non-human algorithms and representations).

Understanding cognition across species, cultures, and hybrid human-AI systems requires expanding both empirical and theoretical frameworks. This final challenge invites cognitive scientists to reconsider foundational assumptions about what constitutes a cognitive system and how cognition should be studied in an increasingly interconnected world.

## Conclusion and call to action

Together, these four challenges unified by Marr's level of analysis highlight both the extraordinary progress of cognitive science and the critical work that lies ahead. Addressing them will require interdisciplinary collaboration, theoretical openness, and methodological innovation. The Cognitive Science section of Frontiers in Psychology provides a uniquely well-suited forum for this effort, welcoming research that is conceptually ambitious, methodologically rigorous, and integrative in scope. We invite researchers from all areas of cognitive science to submit their most innovative theoretical, empirical, and methodological contributions and to help shape the next generation of cognitive science research.
